# Supplementary material for: Advancing Genetic Tools in Streptococcus pneumoniae
Source: Genes (Basel). 2020 Aug 20;11(9):965. doi: 10.3390/genes11090965 (PMC7563404; doi:10.3390/genes11090965)
Supplement: Supplementary file 1 [file genes-11-00965-s001.pdf]

genes

MDPI

Article

Advancing Genetic Tools in *Streptococcus pneumoniae*

Haley Echlin [1] and Jason Rosch [1,*]

[1] Department of Infectious Diseases, St. Jude Children's Research Hospital, Memphis, TN, USA; jason.rosch@stjude.org

* Correspondence: jason.rosch@stjude.org; Tel.: +1-901-595-3408

Received: date; Accepted: date; Published: date

Supplementary Material

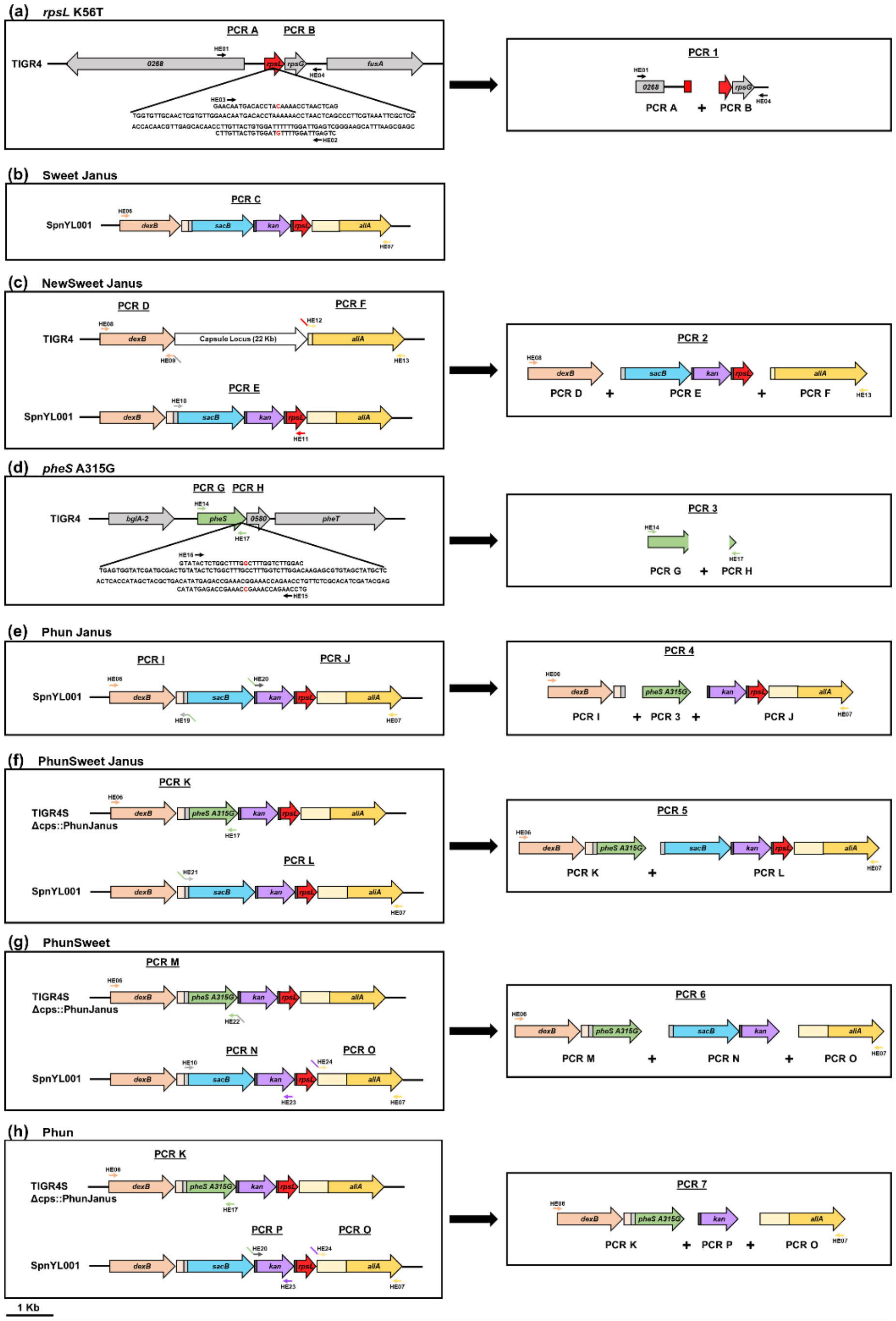

Figure S1. Schematic of PCR primers and template used to generate novel markerless cassettes for allelic replacement of capsule. (a) To confer streptomycin resistance in *S. pneumoniae*, mutation K56T was introduced into *rpsL*. **(b)** Sweet Janus and **(b)** NewSweet Janus were amplified from SpnYL001. **(d)** To confer chlorinated phenylalanine sensitivity, mutation A315G was introduced into *pheS*. **(e)** Phun Janus was generated from *pheS* A315G and SpnYL001. **(f)** PhunSweet Janus, **(g)** PhunSweet, and **(f)** Phun were created using Phun Janus and Sweet Janus as templates. Cassettes were spliced between the genes flanking the capsule loci, *dexB* (tan) and *aliA* (yellow). The cassettes are comprised of genes that confer sucrose sensitivity (*sacB*; blue), kanamycin resistance (*kan*, purple), streptomycin sensitivity (*rpsL*, red), and chlorinated phenylalanine sensitivity (*pheS* A315G, green). The light gray box represents the promoter from Sweet Janus and the dark gray box represents ribosome-binding site. The light-tan box represents the sequence downstream of *dexB* and the light-yellow box represents the sequence upstream of *aliA* from D39. The direction of the arrow indicates the gene orientation. Primers used in generation of each cassette are depicted.

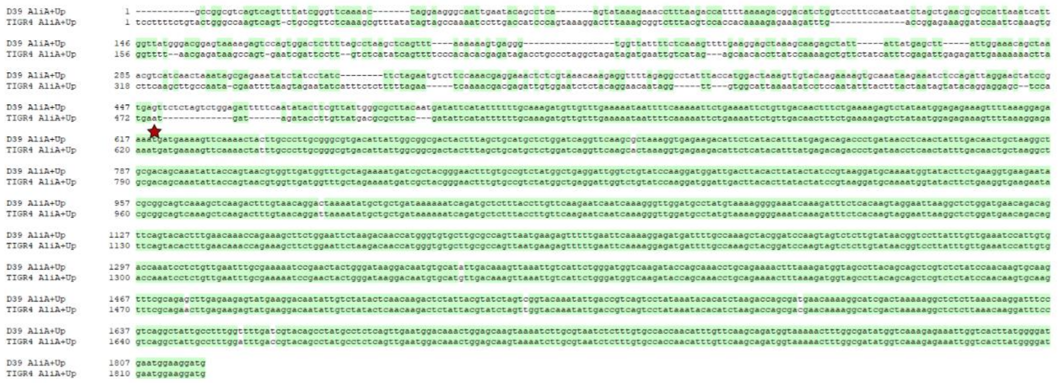

Figure S2. TIGR4 does not share homology with D39 for upstream sequence of *aliA*. Alignment of *aliA* plus upstream sequence from TIGR4 compared to that of D39. Red star indicates start codon of *aliA*.

Table S1. Number and ratio of true-positive to false-positive colonies of allelic replacement of *spxB*. Strain include TIGR4SΔspxB::SweetJanus, D39SΔspxB::SweetJanus, ABCA31SΔspxB::SweetJanus, and CDC001SΔspxB::SweetJanus and were transformed with SOE PCR ΔspxB::Erythromycin. False positives were calculated by subtracting the number of true positive colonies (colonies on plates containing 1 μg/ml erythromycin) from the number of total transformants (colonies on plates containing 800 μg/ml streptomycin plus 10% sucrose). If there were more colonies on plates containing erythromycin than on those containing streptomycin plus sucrose, the number of false positive colonies was considered to be zero. SJ refers to SweetJanus.

Strain	# Total Colonies	# True-positive Colonies	# False-positive Colonies	Ratio
TIGR4SΔspxB::SJ, Clone 1	240	15	225	0.07
TIGR4SΔspxB::SJ, Clone 2	215	15	200	0.08
TIGR4SΔspxB::SJ, Clone 3	120	25	95	0.26
TIGR4SΔspxB::SJ, Clone 4	510	390	120	3.25
TIGR4SΔspxB::SJ, Clone 5	4100	50	4050	0.01
TIGR4SΔspxB::SJ, Clone 6	240	58	182	0.32
TIGR4SΔspxB::SJ, Clone 7	53	25	28	0.89
TIGR4SΔspxB::SJ, Clone 8	290	133	157	0.85
D39SΔspxB::SJ, Clone 1	18	3	15	0.20
D39SΔspxB::SJ, Clone 2	935	925	10	92.50
D39SΔspxB::SJ, Clone 3	68	55	13	4.23
D39SΔspxB::SJ, Clone 4	18000	8000	10000	0.80
D39SΔspxB::SJ, Clone 5	2700	1650	1050	1.57
D39SΔspxB::SJ, Clone 6	470	460	10	46.00
D39SΔspxB::SJ, Clone 7	1043	13	1030	0.01
D39SΔspxB::SJ, Clone 8	1153	950	203	4.68
ABCA31SΔspxB::SJ, Clone 1	95	35	60	0.58
ABCA31SΔspxB::SJ, Clone 2	68	25	43	0.58
ABCA31SΔspxB::SJ, Clone 3	788	533	255	2.09
ABCA31SΔspxB::SJ, Clone 4	8450	9400	0	9400.00
ABCA31SΔspxB::SJ, Clone 5	1280	1875	0	1875.00
ABCA31SΔspxB::SJ, Clone 6	1115	1135	0	1135.00
ABCA31SΔspxB::SJ, Clone 7	75	28	47	0.60
ABCA31SΔspxB::SJ, Clone 8	23	63	0	63.00
CDC001SΔspxB::SJ, Clone 1	3100	2900	200	14.50
CDC001SΔspxB::SJ, Clone 2	3300	3400	0	3400.00
CDC001SΔspxB::SJ, Clone 3	1505	1863	0	1863.00

CDC001SΔspxB::SJ, Clone 4	3600	4100	0	4100.00
CDC001SΔspxB::SJ, Clone 5	983	1170	0	1170.00
CDC001SΔspxB::SJ, Clone 6	3950	1550	2400	0.65
CDC001SΔspxB::SJ, Clone 7	1290	1218	72	16.92
CDC001SΔspxB::SJ, Clone 8	2750	3200	0	3200.00
